# Effects of Heavy Metals/Metalloids and Soil Properties on Microbial Communities in Farmland in the Vicinity of a Metals Smelter

**DOI:** 10.3389/fmicb.2021.707786

**Published:** 2021-08-19

**Authors:** Xuewu Hu, Jianlei Wang, Ying Lv, Xingyu Liu, Juan Zhong, Xinglan Cui, Mingjiang Zhang, Daozhi Ma, Xiao Yan, Xuezhe Zhu

**Affiliations:** ^1^National Engineering Laboratory of Biohydrometallurgy, GRINM Group Co., Ltd., Beijing, China; ^2^School of Metallurgical and Ecological Engineering, University of Science and Technology Beijing, Beijing, China; ^3^GRINM Resources and Environment Tech. Co., Ltd., Beijing, China; ^4^General Research Institute for Non-Ferrous Metals, Beijing, China; ^5^GRIMAT Engineering Institute Co., Ltd., Beijing, China

**Keywords:** heavy metals/metalloids, soil physicochemical properties, microbial communities, high-throughput sequencing, community function

## Abstract

Microorganisms play a fundamental role in biogeochemical cycling and are highly sensitive to environmental factors, including the physiochemical properties of the soils and the concentrations of heavy metals/metalloids. In this study, high-throughput sequencing of the 16S rRNA gene was used to study the microbial communities of farmland soils in farmland in the vicinity of a lead–zinc smelter. Proteobacteria, Acidobacteria, Actinobacteria, Bacteroidetes, and Gemmatimonadetes were the predominant phyla in the sites of interest. *Sphingomonas*, *Gemmatimonas*, *Lysobacter*, *Flavisolibacter*, and *Chitinophaga* were heavy metal-/metalloid-tolerant microbial groups with potential for bioremediation of the heavy metal/metalloid contaminated soils. However, the bacterial diversity was different for the different sites. The contents of heavy metal/metalloid species and the soil properties were studied to evaluate the effect on the soil bacterial communities. The Mantel test revealed that soil pH, total cadmium (T-Cd), and available arsenic played a vital role in determining the structure of the microbial communities. Further, we analyzed statistically the heavy metals/metalloids and the soil properties, and the results revealed that the microbial richness and diversity were regulated mainly by the soil properties, which correlated positively with organic matter and available nitrogen, while available phosphorus and available potassium were negatively correlated. The functional annotation of the prokaryotic taxa (FAPROTAX) method was used to predict the function of the microbial communities. Chemoheterotrophy and airborne chemoheterotrophy of the main microbial community functions were inhibited by soil pH and the heavy metals/metalloids, except in the case of available lead. Mantel tests revealed that T-Cd and available zinc were the dominant factors affecting the functions of the microbial communities. Overall, the research indicated that in contaminated soils, the presence of multiple heavy metals/metalloids, and the soil properties synergistically shaped the structure and function of the microbial communities.

## Introduction

Environmental contamination by heavy metal/metalloid species continues to attract global attention given the persistent nature of such toxic pollutants, which have latent, long-term, cumulative, and non-biodegradability characteristics (Mirzaei et al., 2014; Zhao et al., 2019). Metal smelting activities have resulted in a large number of heavy metal/metalloid species being released into the natural environment, resulting in contamination of soils in the vicinity of smelters (Li et al., 2020b). The accumulation of heavy metal/metalloid species seriously threaten the ecological environment, diversity, functioning of soil microorganisms, food security, and human health.

Soil microorganisms, being the maintainers of the structure and function of the ecosystem, are a key factor for determining soil quality (Hou et al., 2019; Zhou et al., 2019). Soil microorganisms can regulate soil properties, but they are also readily affected by the physicochemical properties of the soils. Studies have shown that microorganisms can significantly promote the circulation of soil nutrients, maintain soil fertility, and improve crop health (Fierer, 2017).

It is known that toxic stress induced by heavy metals/metalloids can seriously affect the abundance and structure of the soil microbial communities and diversity (Zhao et al., 2019) and change ecosystem functions (Bai et al., 2020). For example, heavy metal/metalloid contaminants have affected a variety of functional genes and have altered the ecological functions of soils that were catalyzed by functional groups (Yin et al., 2015; Liu et al., 2018), resulting in a reduction in the microbial diversity and further changing the structure of the soil microbial communities (Liu et al., 2020). Nevertheless, the total concentration of heavy metals/metalloids in contaminated soil over the long-term does not holistically reflect heavy metal/metalloid toxicity (Mondal et al., 2014) because the bioavailability of heavy metals/metalloids can change due to physicochemical interactions with the soil matrix (Wang et al., 2017). Thus, it is desirable to estimate the ecological effects on microbial communities through measurement of the total/available heavy metal/metalloid species.

Previous studies have demonstrated that heavy metal/metalloid pollution causes a change of microbial species, and tolerant microorganisms would replace more susceptible microorganisms and increase in abundance (Guo et al., 2017; Li et al., 2020b). Many microorganisms tolerant to heavy metals/metalloids can remove heavy metals from contaminated soil or convert them to less toxic forms (Zakaria et al., 2007; Khan et al., 2016; Bilal et al., 2018; Qiao et al., 2019). Bioremediation has been considered as a promising eco-friendly and sustainable method for heavy metal pollution remediation (Teng and Chen, 2019). Exploring the microorganisms that are tolerant to heavy metals/metalloids has been helpful for bioremediation studies in heavy metal/metalloid contaminated areas. At present, the data on the relationships between the structure of the soil microbial communities, diversity, and function with respect to total/bioavailable heavy metal/metalloid species are limited (Guo et al., 2017; Tipayno et al., 2018; Zeng et al., 2020).

In addition to being affected by heavy metal/metalloid species, soil microorganisms are also affected by the physicochemical properties of the soil (e.g., pH, organic matter (OM) content, and available N, K, and P) (Tang et al., 2019; Liu et al., 2020; Zeng et al., 2020). Studies have also demonstrated that the microbial communities are affected by the physicochemical properties of the soil (Song et al., 2018; Hou et al., 2019). For example, it has been revealed that soil pH is an important factor that shapes the structure and function of soil microbial communities (Lindsay et al., 2010; Van Der Bom et al., 2018). Moreover, the physicochemical properties of soil may alter the chemical forms of heavy metal/metalloid species, thereby indirectly influencing the structure of the microbial communities (Guo et al., 2017). However, there is little information published on how the structures of the microbial communities, the functions, and the diversity responses to the soil physicochemical properties are affected under long-term stress by heavy metals/metalloids.

In the present research, the main objectives are as follows: (1) to explore the effects of heavy metals/metalloids and the physicochemical properties of soils on the microbial communities in farmland that has been contaminated over the long term with multiple heavy metals/metalloids, and (2) to identify the potential bioremediation microbial groups that are tolerant to heavy metals/metalloids. We hypothesize that the soil microbial communities would be seriously affected by long-term heavy metal/metalloid contamination and that the soil properties could regulate the microbial communities, given that there are heavy metal-/metalloid-tolerant microorganisms that possess a potential bioremediation function. To test this hypothesis, four farmlands around a typical Pb-Zn smelter in Zhuzhou City, China, were sampled, and the heavy metals/metalloids and the soil properties were characterized. Moreover, the diversity, structure, and function of the microbial communities were evaluated. To this end, the intention is to highlight the effects of heavy metals/metalloids and soil properties on the microbial communities in farmland in the vicinity of the smelter and deepen our understanding of the scope and potential for microbial remediation of heavy metals/metalloids in the contaminated soils.

## Materials and Methods

### Site Description and Sampling

**FIGURE 1:**
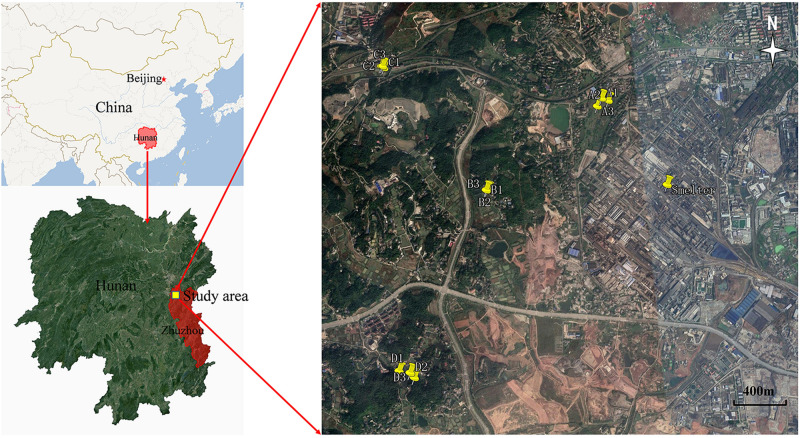
Map indicating the location of four test sites.

The study area is located in farmland adjacent to and west of the Zhuzhou smelter, Zhuzhou, Hunan province, China (Figure 1; 27°51′42″–27°53′13″N, 113°3′30″–113°4′33″E). The area has a typical humid subtropical monsoon climate, with a perennial and dominant north–northwest (NNW) wind except for a southeast (SE) wind in the summer. The Zhuzhou smelter, built in 1956, located in the northwest of Zhuzhou City, is a typical Pb-Zn smelter with a history of more than 60 years. Long-term smelting activities, particularly the unregulated discharge of air particulate dust, has significantly affected the local environment; especially the soil around the smelter is heavily polluted by Pb, Zn, Cd, and As.

Soil samples were collected from farmland at four sites (A, B, C, and D) at various distances from the smelter (Supplementary Table 1). Three separate soil samples were selected at random from the top soil (0–20 cm) at each site, and five subsamples (using the quincunx layout method) were taken from each point and mixed evenly to produce one representative sample (Van Der Bom et al., 2018). All samples (4 sites × 3 sampling points) were placed in sterile bags and refrigerated, and then transported to the laboratory for storage and processing. Each sample (500 g) was homogenized and passed through a 2-mm nylon sieve. One portion was stored at −80°C for DNA extraction and microbial community structural analysis (Luneberg et al., 2018). The other was air dried and stored at 4°C prior to characterization of the physicochemical properties and determination of the heavy metals/metalloids (Li et al., 2020a).

### Heavy Metals/Metalloids

The concentrations of total and available heavy metals (Cd, Pb, and Zn) in the soil samples were determined by inductively coupled plasma optical emission spectrometry (ICP-OES, Agilent Technologies, Malaysia). The soil was digested with 1:2:2 HNO_3_–HClO_4_–HCl (v/v/v) for measurement of the total heavy metals (T-Cd, T-Pb, and T-Zn) (Wang et al., 2014). To determine the available heavy metals (A-Cd, A-Pb, and A-Zn), the diethylene-triamine pentaacetic acid (DTPA) extraction method was used to extract the available heavy metals in soil (Zahedifar et al., 2016). For the concentrations of total arsenic (T-As) determination, samples were pretreated by aqua regia digestion, and the concentrations of available arsenic (A-As) was extracted with 0.5 mol/L disodium hydrogen phosphate prior to measurement with an atomic fluorescence spectrophotometer (AFS-820, Beijing Jitian Instrument Co. Ltd, China) (Wu et al., 2019). The Nemeiro pollution indices (*P*_*N*_) (Pan et al., 2020) and the geoaccumulation indices (*I*_*geo*_) (Wang et al., 2019) were used to evaluate the comprehensive pollution and single pollution of heavy metals/metalloids, respectively.

### Physiochemical Properties of Soil

The soil pH was measured at a solid–liquid ratio of 1:2.5 (w/v) with a calibrated pH meter (Orien3-star, Thermo Fisher Scientific, Singapore), and OM in soil was determined by potassium dichromate oxidation titration (Liu et al., 2014). The soil available nitrogen (AN) was measured with the NaOH hydrolysis method (Gianello and Bremner, 2008), available phosphorus (AP), and available potassium (AK) were measured by a spectrophotometer at 700 nm (TU-1810, Purkin, China) and a flame photometer (AA-6300, SHIMADZU, Japan) (Song et al., 2018), respectively.

### Microbial Communities and Diversity

The total genomic DNA in soil samples was extracted with the E.Z.N.A. bacterial DNA kit (OMEGA, M5635-2). The bacterial 16S rRNA genes of the V3–V4 region were amplified (PCR) using the 341F (5’-CCTACGGGNGGCWGCAG-3’) and 805R (5’-GACTACHVGGGTATCTAATCC3’) primers (Schneider et al., 2017). Sequencing was carried out using the Illumina MiSeq platform (v.2.4.60.8; Illumina, CA, United States). The raw sequence results were processed and analyzed with QIIME (v.1.8.0) and Mothur (v.1.30.1) (Kasemodel et al., 2019). To obtain effective sequences, the reads were subjected to quality control with FLASH (v.1.2.3) (Guo et al., 2017; Kasemodel et al., 2019). Representative sequences were clustered at a similarity level of 97% of operational taxonomic units (OTUs) (Lin et al., 2019). The OTU taxonomies were determined using the RDP Classifier script and were determined at a similarity level of 97% at species-level taxonomies. The relative abundance of each taxon in the community was calculated by comparing the sequence number of a specific taxon with the total sequence number of the selected samples. Functional annotation of prokaryotic taxa (FAPROTAX) was used to predict the function of the microbial communities in the soils of the study areas (Li et al., 2020a).

### Data Analysis

Statistical analysis of all bacterial sequences was performed using the R software package (vegan, v.3.2.3) (Liu et al., 2020). The relative abundance and diversity of the microbial communities were reflected by the Alpha diversity and the unweighted pair group method with arithmetic mean (UPGMA) cluster analysis, which were calculated by QIIME (v.1.8.0). Principal coordinates analysis (PCoA), Mantel tests, Spearman’s correlation analysis, and the heatmap of environmental factors and microbial communities were carried out with the R software package (vegan, ggplot2, v.3.2.3). A comparison of the physicochemical properties; the total concentration of As, Cd, Pb, and Zn; and the available metal concentrations for the four discrete sites (A, B, C, and D) were investigated using one-way ANOVA, and comparison of means was conducted by Duncan multiple range tests. Most statistical analyses were performed using SPSS 25, and a part of the statistics was performed using Origin 2017.

## Results

### Physiochemical Properties of Soils and Heavy Metal Concentrations

**FIGURE 2:**
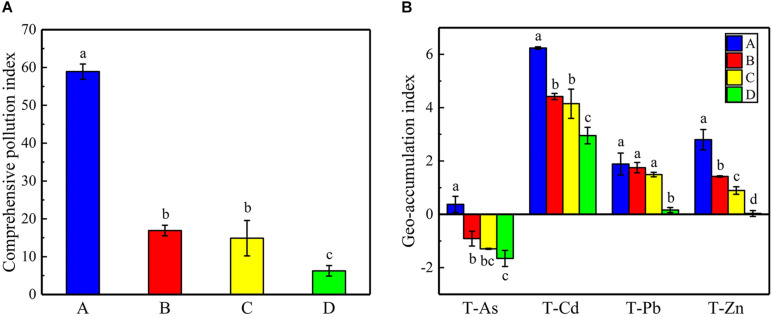
**(A)** The comprehensive pollution indices for heavy metals/metalloids at the sampling sites and **(B)** the geoaccumulation indices for T-As, T-Cd, T-Pb, and T-Zn at the sampling sites. Error bars indicate standard error of the mean (*n* = 3). The symbols represent different levels of significance at *p* < 0.05 for the sites.

**FIGURE 3:**
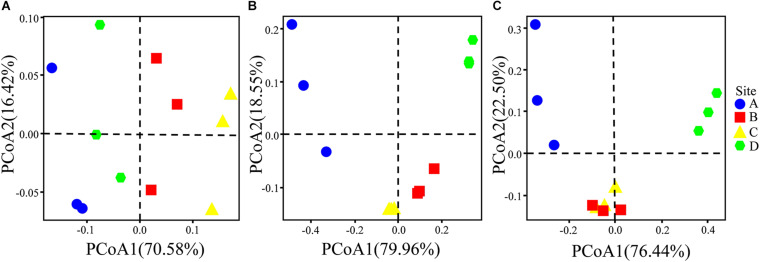
PCoA plots depicting **(A)** the physicochemical properties of the soils and **(B)** total heavy metals/metalloids in the soils, and **(C)** the available heavy metals/metalloids in the soils based on the Bray–Curtis distances at the sampling sites.

The physicochemical properties of the soils and the total/available content of heavy metals/metalloids in the study regions were measured (Supplementary Table 2). The results show that the soil pH was moderately acidic at sites B, C, and D (4.66 ± 0.66 to 5.20 ± 0.53), whereas site A had neutral pH (7.30 ± 0.15). The contents of heavy metals/metalloids in the four areas were higher than the screening value for risk of soil pollution for agricultural dryland (GB 15168-2018; Supplementary Table 3). The comprehensive pollution indices were used to evaluate the contamination levels for the heavy metals/metalloids at the four sites (Figure 2A). The results showed that the soils may be classified as heavily polluted based on the grading standard for the degree of comprehensive soil pollution (Supplementary Table 4); furthermore, sites A and D were the most heavily and the least heavily polluted, respectively. There was no significant difference between the indices of sites B and C. Furthermore, the geoaccumulation indices (I_*geo*_) were calculated based on the contents of As, Cd, Pb, and Zn. It was found that the I_*geo*_ of the soils varied with the site and the nature of the heavy metals/metalloids (Figure 2B). For example, all sites could be classified as being moderately to extremely polluted with Cd, while only site A could be classified as polluted with As (light to moderate pollution; according to Supplementary Table 5). In addition, PCoA indicated obvious differences in the total/available content of heavy metals/metalloids and in the physicochemical properties of the soils across the study sites (Figure 3).

### Differences Among Bacterial Communities at Different Sampling Sites

**FIGURE 4:**
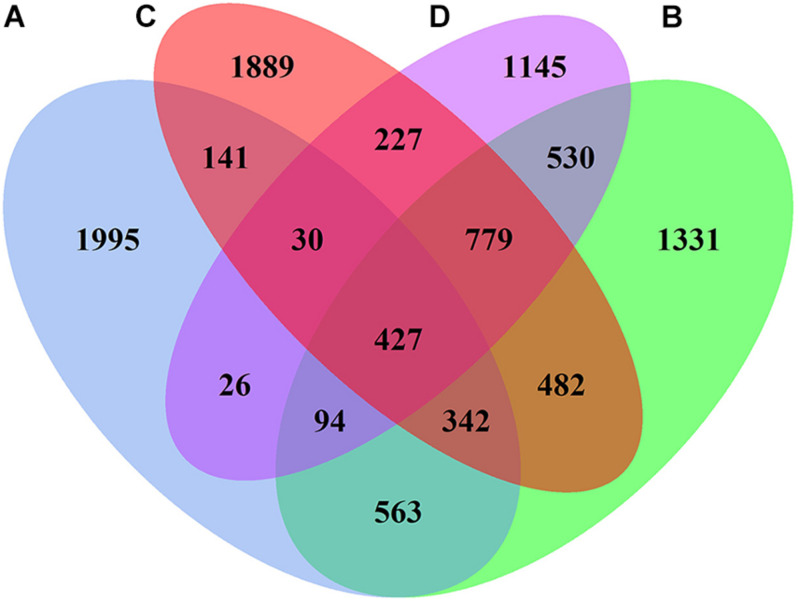
Venn diagram displaying the number of shared bacterial OTUs between the soil samples collected at different sites.

**FIGURE 5:**
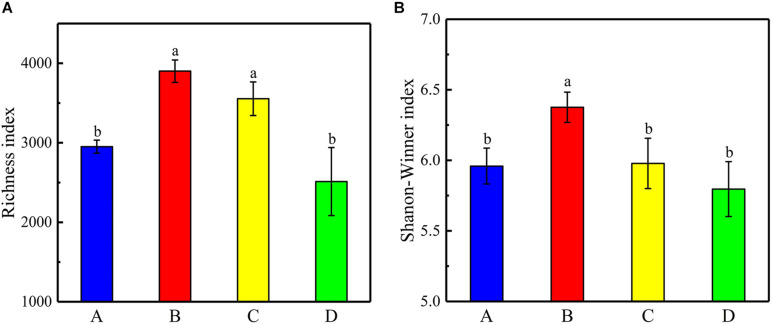
**(A)** Shannon–Winner indices and **(B)** the richness indices of OTUs for all samples at the different sites. Error bars indicate standard error of the mean (*n* = 3). The symbols represent different levels of significance at *p* < 0.05 for the sites.

The 16S rRNA genes in 12 samples (4 sites × 3) were detected using the Illumina MiSeq300 platform, where a total of 731,888 qualified sequences were obtained with a range of 53,675–83,547 sequences in each sample. The Venn diagram of bacterial OTUs showed that the specific OTU richness at the different sites generally followed the number of sequences (in descending order): A > C > B > D (*p* < 0.05, Figure 4). Moreover, there were 427 core OTUs in samples from all four sites, and soil samples from sites A, B, C, and D contained 1,995, 1,331, 1,889, and 1,145 unique OTUs, respectively. The OTU richness diversity indices and Shannon–Winner indices were used to evaluate the diversity and richness of the soil microbial communities. The results showed that the OTU richness indices were significantly higher at sites B and C than those at sites A and D, and the Shannon–Winner indices at site B were significantly higher than at other sites (Figure 5).

**FIGURE 6:**
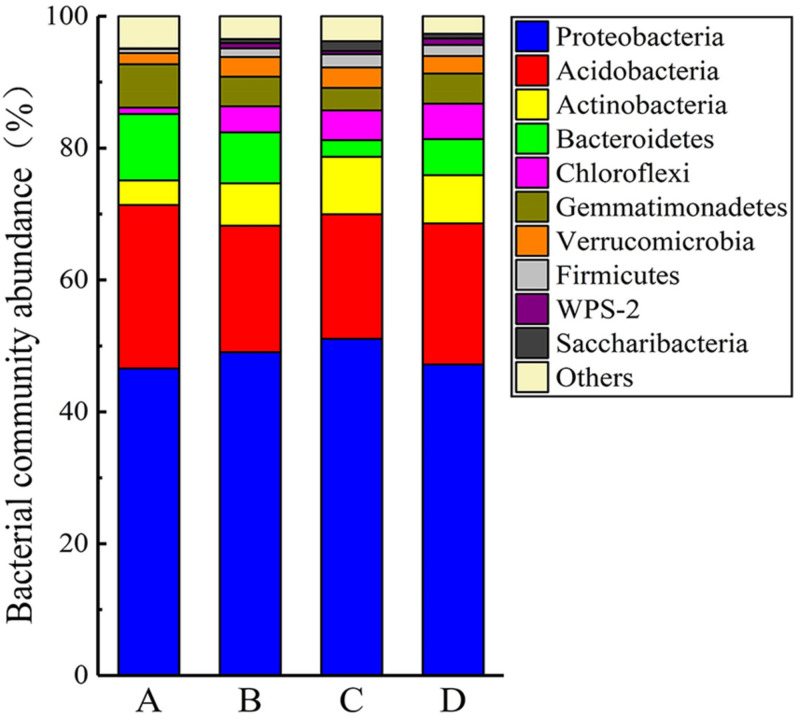
Composition of bacterial communities at the four sampling site, showing the 10 most abundant phyla (with relative abundance > 1% of total reads). Minor phyla (with relative abundances < 1%) were pooled together.

The PCoA analysis based on the Bray–Curtis distance explained approximately 61.89% of the microbial community composition variation (first and second dimensions were 41.52 and 20.37%, respectively; Supplementary Figure 1). Soil heavy metal/metalloid contamination and soil properties clearly affected the composition of the soil bacterial communities, as reflected by the PCoA analysis. Taxonomic classification of all the OTUs identified 32 different phyla. The compositions of the bacterial communities (on the phylum level) at the four different sites are shown in Figure 6. The predominant phyla across all the sites were Proteobacteria, Acidobacteria, Bacteroidetes, Actinobacteria, and Gemmatimonadetes, accounting for more than 83% of the bacterial sequences from each of the soils. The most frequently detected bacterial phyla in the analyzed samples were Proteobacteria (46.34–50.48%), Acidobacteria (18.66–24.66%), Bacteroidetes (2.49–10.02%), Actinobacteria (3.37–8.64%), Gemmatimonadetes (3.38–6.54%), Chloroflexi (0.97–5.35%), Verrucomicrobia (1.70–3.06%), and Firmicutes (0.63–1.99%). Besides, we found that there were archaea with a low abundance of 0.13% in the soil samples. With increasing soil pollution, the proportions of Acidobacteria, Bacteroides, and Bacillus increased, while the relative abundances of Proteobacteria and Actinomyces decreased. In addition, the proportions of the less abundant phyla Chloroflexi, Verrucomicrobia, and Firmicutes negatively correlated with the contamination level. Unclassified bacteria accounted for large proportions (21.77–31.21%) of genera at all four sites. A similar situation was observed in a previous investigation on the effect of heavy metal/metalloid pollution on soil microbial community in smelting site, which reported a mean relative abundance of unclassified genera to be 26.80% (Li et al., 2020a). Other major genera included *Sphingosinicella* (3.80–14.10%), *Sphingomonas* (3.10–10.83%), *Gemmatimonas* (3.38–6.54%), *Gp1* (0.07–7.46%), *Gp6* (1.85–9.47%), *Gaiella* (0.85–3.43%), *Gp3* (1.11–3.07%), *Gp4* (0.13–6.85%), *Gp2* (0.01–2.79%), *Lysobacter* (0.20–2.07%), *Flavisolibacter* (0.11–1.19%), and *Chitinophaga* (0.01–1.56%) (Supplementary Figure 2A). The difference in the average abundance of the top 30 microbial genera at each site can be clearly seen from inspection of Supplementary Figure 2B. There were clear differences (*p* < 0.05) in the abundance of *Sphingosinicella*, *Sphingomonas*, *Gp1, Gp6*, *Gaiella*, *Gp3, Gp4*, and *Chitinophaga* among the four sites. The dominant genera of *Sphingomonas*, *Gp6*, *Gp4*, *Lysobacter*, *Flavisolibacter*, and *Chitinophaga* increased with increasing soil contamination, while *Sphingosinicella*, *Gp1*, *Gaiella*, and *Gp3* decreased with increasing soil contamination.

### Functional Groups Within the Soil Prokaryotic Communities

**FIGURE 7:**
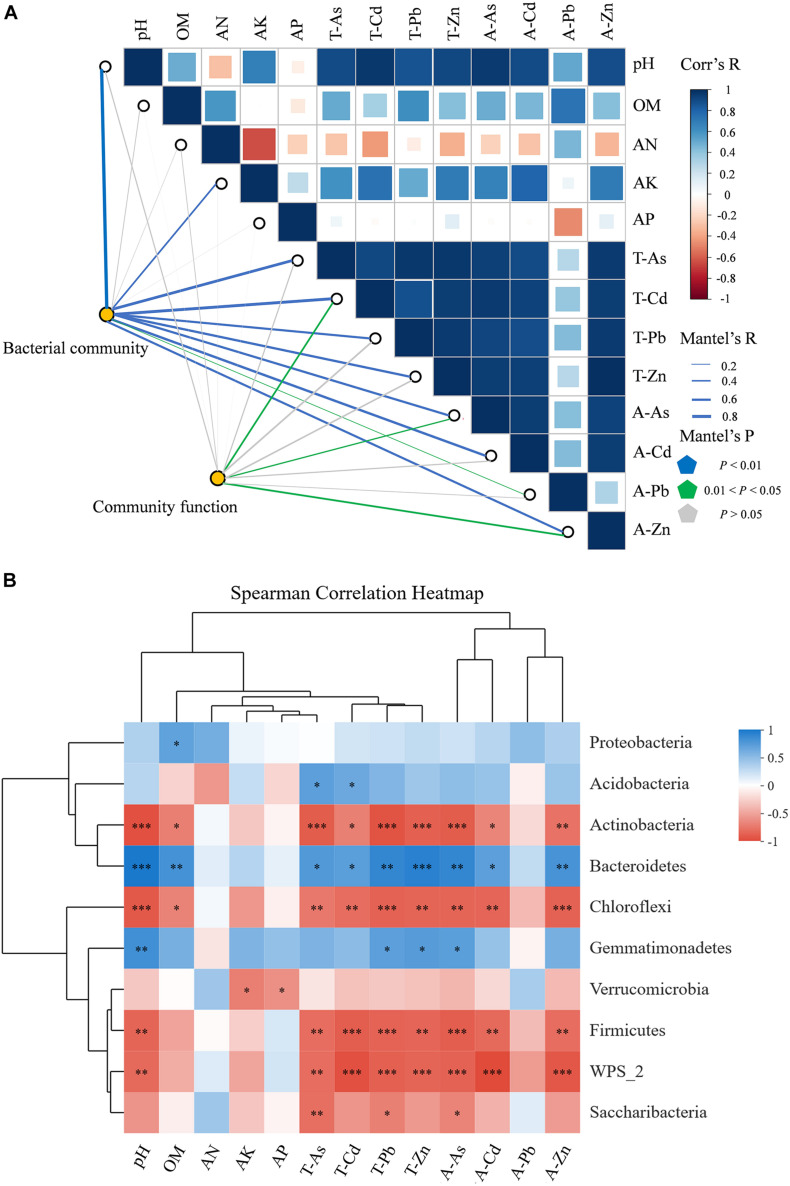
**(A)** Pairwise comparisons of ecological drivers, with a color gradient denoting the Spearman’s correlation coefficients. The structure of the bacterial communities was correlated with each ecological driver by partial Mantel tests, as well as by microbial function. Curve width and color represent the correlation coefficients of the partial Mantel tests; blue in the figure indicates *p* < 0.01, purple indicates 0.01 < *p* < 0.05, and gray indicates p > 0.05. **(B)** Heatmap of Spearman’s rank correlation coefficients between the four sites for the relative abundances of bacterial phyla, including the relative abundances (%, *n* = 3) of the top 10 microbial phyla. Note: Horizontal row represents physiochemical properties of the soils and the metals/metalloid information; the vertical row represents information on the abundance of the microbial communities; red represents negative correlation, blue represents positive correlation, a darker color indicates a higher correlation; the p value is the correlation test result; *p < 0.05, **p < 0.01, ***p < 0.001. T-As, total concentration of arsenic; T-Cd, total concentration of cadmium; T-Pb, total concentration of lead; T-Zn, total concentration of zinc; A-As, available content of arsenic; A-Cd, available content of cadmium; A-Pb, available content of lead; A-Zn, available content of zinc.

We used FAPROTAX to map the prokaryotic clades to metabolic or other ecologically relevant functions. Among the 90 functional groups in the FAPROTAX database, 52 groups (57.8%) were present in at least one of the samples (Supplementary Table 6). The correlation functions for FAPROTAX showed that chemoheterotrophy (average 39.70%) and aerobic chemoheterotrophy (average 37.60%) were the two main potential metabolic types. Heavy metals/metalloids and the physicochemical properties of the soils associated with functional groups were identified by the Mantel test (Figure 7A). The results suggested that the functional groups were correlated with T-Cd and A-Zn (*R* = 0.454, *p* < 0.05; *R* = 0.478, *p* < 0.05). Furthermore, Spearman’s correlation analysis revealed that these functional groups included taxa (Supplementary Table 7) associated with processes in the biogeochemical cycling of C and N.

### The Association of the Microbial Communities in the Soils and Environmental Parameters

To assess the relative effect of environmental parameters (five physicochemical properties of soil including pH, OM, AN, and AP; four metals/metalloids: T-As, T-Cd, T-Pb, T-Zn, A-As, A-Cd, A-Pb, and A-Zn) on the abundance and diversity of bacterial communities in the metal/metalloid contaminated soil, the Mantel test was conducted (Figure 7B). The results showed that, except for OM, AN, and AP, the environmental parameters had a significant influence on the microbial communities (*p* < 0.05). Among all the environmental factors, the pH (*R* = 0.882, *p* < 0.001), T-Cd (*R* = 0.770, *p* < 0.001), and A-As (*R* = 0.734, *p* < 0.001) had the strongest correlation with the microbial community composition. In addition, there was also a significant correlation between the bacterial communities and A-Cd, T-Zn, T-As, A-Zn, T-Pb, A-Pb, and AK in the soil (*p* < 0.01).

The heatmap based on Spearman’s correlation analysis was used to study the relationships among the environmental parameters and the relative microbial abundance on the phylum level (1%) (Figure 7B). The abundance of the predominant phylum Proteobacteria was positively correlated with OM (*p* < 0.05). The other predominant phylum Acidobacteria was positively correlated with T-As and T-Cd (*p* < 0.05). Actinobacteria and Chloroflexi were negatively correlated with pH, OM, T-As, T-Cd, T-Pb, T-Zn, A-As, A-Cd, and A-Zn, while Bacteroidetes was the opposite. Gemmatimonadetes exhibited a positive correlation with pH, T-Pb, T-Zn, and A-As (*p* < 0.05). On the genus level, the heatmap showed correlation between microorganisms and the environmental factors as depicted in Figure 3. *Sphingomonas* exhibited a positive correlation with pH and AK (*p* < 0.05), and *Gemmatiomonas* exhibited a positive correlation with pH (*p* < 0.05). *Gp1*, *Gaiella*, and *Gp3* were negatively correlated with pH and most heavy metals/metalloids, while *Gp6* and *Gp4* exhibited a positive correlation with pH and most heavy metals/metalloids.

## Discussion

### Physicochemical Properties of the Soils and Characteristics of Metal/Metalloid Pollution

In this study, it was found that the physicochemical properties of the soils and the concentrations of heavy metals/metalloids differed for each site (Supplementary Table 2). The pH values of all samples ranged from 4.66 ± 0.66 to 7.30 ± 0.15, and this finding was similar to that for a previous study on farmland soil (Li et al., 2011). The soil pH was acidic at sites B, C, and D, while the pH at site A was neutral, being maintained by the long-term deposition of a lime powder (Li et al., 2011). The OM in the soils at sites A, B, and C were clearly higher than that of site D, and this finding was similar to that of a previous study (Shen et al., 2016). The content of AK at site B was lower than those at other sites; however, there was no significant differences in the content of AN and AP at the four sites.

The concentrations of all heavy metals/metalloids at the four sites markedly exceeded the screening limit value for the pollution risk of soil in agricultural dryland (GB 15168-2018; Supplementary Table 3), a situation that represented serious pollution, presenting risks for the environment as well as human health. In this study, the content of metals/metalloids, the comprehensive pollution indices, and the geoaccumulation indices typically decreased with increasing distance from the Pb-Zn smelter, a finding that was reported previously (Li et al., 2011; Lei et al., 2016; Shen et al., 2017). The differences between site C and site D reflected the fact that site C was in the main direction of the wind, which increased the metal pollution at the site. Moreover, due to the influence of the smelting activities involving Pb and Zn, the elements As, Cd, Pb, and Zn have been widely reported as the main pollutants, especially Cd, Pb, and Zn (Li et al., 2011, 2015, 2020b; Shen et al., 2016; Tipayno et al., 2018). However, the total heavy metal/metalloid concentrations in long-term contaminated soils may not holistically reflect the heavy metal/metalloid toxicity (Mondal et al., 2014), because the bioavailability of heavy metals/metalloids might change according to their physicochemical interactions with the soil matrix (Wang et al., 2017). Thus, it is necessary to estimate the ecological effects defined by the availability of heavy metals/metalloids.

The Spearman’s correlation analysis (Figure 7A) indicated that soil pH exhibited a significantly positive correlation with heavy metals/metalloids (*p* < 0.01) and OM (*p* < 0.05). Further research has found that pH was positively correlated with the proportion of A-As in the total As, while there were negative correlations with the ratios of A-Cd, A-Pb, and A-Zn (Supplementary Table 8). It has been recognized that a decrease in the soil pH affects the solubility of heavy metals (e.g., Cd, Pb, and Zn), thus increasing their mobility and bioavailability (Wang et al., 2006; Ma et al., 2020). In contrast, as the soil pH decreases, the anions containing As would tend to be adsorbed by the positively charged iron oxide, thereby reducing the mobility and hence the toxicity of As (Jain et al., 1999). Previous studies have confirmed that OM is involved in the adsorption, complexation, and precipitation of the heavy metals/metalloids, thereby weakening the bioavailability of heavy metals/metalloids (Bolan et al., 2014; Mazurek et al., 2017). The same experimental results have also been confirmed in our study.AN, AP, and AK did not show significant positive correlations with heavy metals/metalloids.

### Effects of the Soil Physicochemical Properties on the Microbial Community Composition

The microbial abundance and diversity of the soil are affected by various physicochemical properties of the soil (Liu et al., 2020). In this work, soil pH was not significantly correlated with the bacterial OTU richness indices and the Shannon–Winner indices (Supplementary Table 9). A similar study reported that the soil pH was not correlated with alpha diversity (Zarraonaindia et al., 2020). In addition, our results revealed that soil pH was a relevant factor in shaping the structure of the bacterial communities of the soils (Figure 7A), which is consistent with the work of Guo et al. (2017), who reported the soil pH was the most critical factor affecting the structure of the bacterial communities. Especially, there was no significant correlation between pH and the dominant bacterial phyla Proteobacteria and Acidobacteria, and the relative abundance of Acidobacteria at site A (neutral pH) was greater than at other sites. However, on the genus level, the dominant genera *Sphingomonas* and *Lysobacter* of the Proteobacteria, and the dominant genera *GP1*, *GP6*, *GP3*, *GP4*, and *GP2* of the Acidobacteria had a significant correlation with pH, positive or negative (Supplementary Figure 3). The above-mentioned inconsistencies in correlations between pH and phylum abundances might be due to the metabolic diversity of species belonging to the phyla Proteobacteria and Acidobacteria. As for site A, the dominant bacteria genera *GP6* and *GP4* were significantly positively correlated with pH, and the relative abundances were significantly higher than in samples from other sites. Previous studies confirmed that the relative abundance of Acidobacteria increased in soils with higher pH in heavy metal-contaminated soils (Pan et al., 2020). Moreover, Wang et al. (2020a) found that the dominant genera were identified as *GP6* and *GP4* in areas with a pH around 8.0. This may explain why the Acidbacteria phylum at site A was more abundant. Besides, OM was positively correlated with the bacterial richness indices; however, it was negatively correlated with some processes involving the biogeochemical cycling of C. These correlations may reflect the fact that OM is the main source of C and N for microorganisms, hence the direct link between OM and the number and/or types of microorganisms (Xie et al., 2017).

The nutrients in the soil are closely involved in the biogeochemical cycling of elements mediated by soil microorganisms (Bai et al., 2020). A recent study demonstrated through statistical analysis that A-N was positively correlated with the abundance and diversity of bacterial communities in Cd-contaminated soils (Liu et al., 2020). Similarly, in the present study, AN was shown to have a significantly positive correlation with the bacterial richness indices and the Shannon–Winner indices (Supplementary Table 9). Moreover, some functional groups associated with N cycling showed positive correlations with AN. Interestingly, AK was found to be negatively correlated with the bacterial Shannon indices. This finding is consistent with a previous report (Pan et al., 2020). This negative correlation for AK may be the reason why the richness and diversity at site B were higher than those of site A, as the AK in site A is greater than that at site B (Supplementary Table 2). A previous study confirmed that AP was negatively correlated with the microbial abundance and the diversity in Cd-contaminated soil (Liu et al., 2020), which is consistent with our results. Based on the above studies, the lower OM, AN, and higher AP contents may be the reason for the lowest abundance and diversity at site D, which had the lowest heavy metal/metalloid pollution among the four tested sites.

The effect of the physicochemical properties of the soils on the different bacterial phyla and genera clearly varies (Pan et al., 2020). Significant correlations between the abundance of the most dominant taxa (8 out of 10 major phyla and 20 out of 30 major genera) with at least one soil parameter were observed in this study. pH was the predominant factor for the specific bacteria phyla and genera, followed by OM. AN, AP, and AK showed no significant correlation with the predominant bacterial phyla. It is believed that the relative abundance of Bacteroidetes was positively correlated with pH, while Actinobacteria was negatively correlated (Zarraonaindia et al., 2020). A similar pattern was observed in the present study. Moreover, our results showed highly positive correlations between pH and the relative abundances of dominant Sphingomonas and Gemmatimonas. The most dominant Proteobacteria was positively correlated with OM in the soils examined. One possible reason for this is that Proteobacteria have a complex metabolism, utilizing various forms of OM as sources of C, N, and energy (Bouskill et al., 2010). In addition, also the relative abundance of Bacteroidetes was clearly positively correlated with OM. This may be due to the fact that Bacteroides are usually eutrophic and dominant in soils with large amounts of labile organic carbon (Will et al., 2010).

### Effects of Metal/Metalloid Pollution on the Function of Microbial Communities in Soils

Long-term heavy metal/metalloid stress would tend to increase the abundance of tolerant microorganisms and decrease the abundance of sensitive microorganisms, thereby affecting the abundance and structure of the microbial communities and diversity (Zhao et al., 2019). A study revealed the bioavailable metals/metalloids had an important effect on the diversity and composition of bacterial communities (Zhao et al., 2019). In this study, the structure of the microbial communities in heavy metal/metalloid polluted soil changed significantly with the degree of pollution (Supplementary Figures 2, 4, 6, 7B). Moreover, the results for the Mantel tests revealed that the heavy metals/metalloids T-Cd, A-As, and A-Cd played an important role in the establishment of the microbial communities (Figure 7A). However, from all the metals/metalloids, only A-Pb was significantly positively correlated with bacterial richness indices and the Shannon–Winner indices (*p* < 0.05, Supplementary Table 9).

It is worth noting that seven heavy metal/metalloid parameters significantly correlated, either positively or negatively, with 8 out of 10 most abundant phyla at all four sites (Figure 7B). In the study by Janssen (2006), the dominant phyla in the healthy soil samples were Proteobacteria (39%), followed *by Acidobacteria* (20%) and Actinomycetes (13%); the remaining 28% of the communities consisted of Verrucomicrobia, Bacteroidetes, Chloroflexi, planctomyctes, and Gemmatimonadetes (all ranging from 2 to 7%). The results for the soil samples in the present study clearly differed from those of the microbial communities in healthy soils reported by Janssen. Proteobacteria (46.34–50.48%) were one of the most dominant phyla, followed by Acidobacteria (18.66–24.66%). Other abundant phyla were Bacteroidetes, Actinobacteria, and Gemmatimonadetes (all ranging from 2.49 to 10.02%). The results obtained from the long-term heavy metal/metalloid-polluted sites are consistent with results obtained in independent studies in similar smelter areas (Li et al., 2017, 2020b; Schneider et al., 2017; Bai et al., 2020). According to reports, the phylum Proteobacteria contain most of the heavy metal-resistant genes, followed by Actinobacteria and Bacteroidetes, as revealed by metagenome sequencing (Yan et al., 2020). This may explain high relative abundances of Proteobacteria in samples analyzed in this study. However, we found that the correlation between Proteobacteria and heavy metals/metalloids was not significant. This may be due to the fact that the long-term heavy metal/metalloid contamination had altered the soil microbial communities in such a way that the communities were in a stable state (Beattie et al., 2018).

On the genus level, differences (*p* < 0.05) were observed among mean abundances of 15 out of 30 genera at the four sites (Supplementary Figure 2B); and the heatmap for Spearman’s correlation analysis showed that 21 bacterial genera were clearly affected by at least one heavy metal/metalloid (Supplementary Figure 4). The predominant bacterium *Sphingosinicella* exhibited no obvious correlation with heavy metals/metalloids. While other predominant bacteria *Sphingomonas* and *Gemmatimonas* were moderately correlated with heavy metals/metalloids except A-Pb. Similar to this study, some researchers found that *Sphingomonas* was highly resistant to arsenic and antimony (Li et al., 2021b), and another study proved that *Gemmatimonas* was more abundant in heavily polluted areas (Guo et al., 2017).

Long-term exposure to heavy metals/metalloids can also affect the functional characteristics of the communities (Tipayno et al., 2018). According to previous reports, heavy metal stress can affect a variety of functional genes and change the soil ecological functions that are catalyzed by functional groups (Yin et al., 2015; Liu et al., 2018). Moreover, it has been reported that 30 functional groups in heavy metal-contaminated soil at a smelter site were affected by heavy metals/metalloids (Bai et al., 2020). Similarly, our results indicated that there were eight functional groups that were significantly correlated with different heavy metals/metalloids, and the two main functional groups (chemoheterotrophy and aerobic chemoheterotrophy) exhibited remarkable negative correlation with heavy metals/metalloids except A-Pb (Supplementary Table 7).

### Combined Effects of the Heavy Metals/Metalloids and the Soil Properties on the Microbial Communities

In this study, Mantel tests were used to identify the respective contributions made by the heavy metals/metalloids and physicochemical properties of the soil to the bacterial communities. The Mantel tests revealed that pH (*R* = 0.882, *p* < 0.001), T-Cd (*R* = 0.770, *p* < 0.001), and A-As (*R* = 0.734, *p* < 0.001) played a key role in the establishment of the microbial communities (Figure 7A). The soil pH may influence the microbial communities in the soil contaminated by complex heavy metals/metalloids through two possible pathways. First, any change in pH will put pressure on single-celled organisms (Fierer and Jackson, 2006). Second, soil pH can directly affect the chemical forms of heavy metals/metalloids in the soil and indirectly affect the adsorption interactions between heavy metals/metalloids and the soil microorganisms (Jiang et al., 2019; Ma et al., 2020). Furthermore, heavy metals/metalloids in soil and soil pH were found to be the dominant factors affecting the microbial communities in soils (Li et al., 2020b; Zeng et al., 2020). In this study, the soil pH and the heavy metals/metalloids were found to shape the microbial communities synergistically, especially with the same significant positive or negative correlations for predominant phyla/genera, that is, for the conditions with higher contents of heavy metals/metalloids and pH, the abundances of Acidobacteria, Bacteroidetes, and Gemmatimonadetes, and the genera *Sphingomonas*, *Gemmationas*, *Gp6*, *Gp4*, *Lysobacter*, *Flavisolibacter*, and *Chitinophaga* increased. Therefore, the soil bacterial communities were shaped as a result of the synergistic effects of the heavy metals/metalloids in the soils and the physicochemical properties of the soils.

To date, there is limited information on the responses of the microbial communities in soils to long-term pollution with heavy metals/metalloids at the ecological level. Mantel tests revealed that T-Cd (*R* = 0.454, *p* < 0.05) and A-Zn (*R* = 0.478, *p* < 0.05) were the key factors affecting the functionality of the soil microbial communities (Figure 7A). Spearman’s correlation analysis shows that heavy metals/metalloids and the physicochemical properties of the soils had a remarkable effect on multiple biogeochemical cycling, especially on the inhibition of chemoheterotrophy and airborne chemoheterotrophy (Supplementary Table 7). However, a recent study at a smelter site showed that multiple functional groups related to various biogeochemical processes were selectively affected by different heavy metals/metalloids and the physicochemical properties of the soils, while most of the main functional groups associated with chemoheterotrophy and aerobic chemoheterotrophy were not significantly affected (Bai et al., 2020). The conflicting results can be attributed to the changes in the environmental parameters for the different study areas.

### Bioremediation Potential of Bacterial Groups Identified in Soils Long-Term Contaminated With Heavy Metals/Metalloids

In this study, *Sphingosinicella* and *Sphingomonas* were the most abundant genera belonging to Proteobacteria. Proteobacteria contain species that have been widely reported to be involved in heavy metal resistance, biosorption, or biotransformation. *Sphingosinicella* have been reported to be resistant to As (Qiao et al., 2019). Previous studies have shown that *Sphingomonas* were widespread and relatively abundant in heavy metal/metalloid-contaminated soils due to long-term smelting and mining operations (Guo et al., 2017; Wang et al., 2017; Kasemodel et al., 2019; Li et al., 2020b). Moreover, *Sphingomonas* have been shown to play a potential role in the bioremediation of various heavy metals/metalloids, for example, Cd (Khan et al., 2016), Zn (Baskin et al., 2014), As (Kinegam et al., 2008), Cu (Wang et al., 2010), and Pb (Chen, 2011). Furthermore, it has been demonstrated that heavy metal-resistant genes in *Sphingomonas*, such as the czc operon, make *Sphingomonas* show resistance to Co, Zn, and Cd by controlling the metabolic process, and copA is the resistance gene for Cu (Asaf et al., 2018, 2020). Therefore, *Sphingomonas* has great potential for bioremediation of heavy metals/metalloids in contaminated soils. In addition, *Lysobacter* has a significant positive correlation with most heavy metals/metalloids (Supplementary Figure 3). *Lysobacter* belongs to the phylum Proteobacteria, which have been reported to be tolerant to Hg, As, Cr, Pb, Co, Cu, and Zn (Brito et al., 2013; Puopolo et al., 2016; Yaseen et al., 2018; Wang et al., 2020b). Yaseen et al. (2018) have confirmed that *Lysobacter novalis* can reduce A-Pb in soil by producing biosurfactants. It is worth noting that *Gemmatimonas* of the phylum Gemmatimonadetes, and *Flavisolibacter* and *Chitinophaga* of the phylum Bacteroides also exhibit heavy metal/metalloid resistance in this work. For example, *Gemmatimonas* is tolerant to As (Li et al., 2021a), *Flavisolibacter* is tolerant to Pb and Zn (Kasemodel et al., 2019), and *Chitinophaga* is tolerant to Ni, Cu, Zn, and Cd (Chanda et al., 2017). The interaction between bacteria and heavy metals is complex, making it a challenge to understand the basic mechanisms. Thus, the mechanisms of metal transformations by these bacteria need to be further studied in order to be able to fully exploit the full potential for bioremediation in heavy metal/metalloid contaminated soils.

## Conclusion

It has been demonstrated that microbial richness and diversity in soil samples are regulated mainly by the physicochemical properties of the soil, both parameters exhibiting positive correlation with OM and AN, while AP and AK were negatively correlated. The structure and function of microbial communities in contaminated soils were affected by the physicochemical properties and the concentrations of heavy metals/metalloids. Among the factors, pH, T-Cd, and A-As played an important role in the establishment of the structures of the microbial communities, and T-Cd and A-Zn were found to be the key factors affecting the functions of the microbial communities. In addition, it was found that *Proteobacteria, Acidobacteria, Actinobacteria, Bacteroidetes, and Gemmatimonadetes* were the predominant phyla in long-term heavy metal/metalloid-contaminated soils. The research further revealed that *Sphingomonas*, *Gemmatimonas*, *Gp6*, *Gp4*, *Lysobacter*, *Flavisolibacter*, and *Chitinophaga* were highly tolerant to metals/metalloids. All these groups, except for GP6 and GP4, have a potential for bioremediation of heavy metal/metalloid pollution. Chemoheterotrophy and airborne chemoheterotrophy, key functions of the microbial communities, were inhibited by heavy metals/metalloids and the soil pH. In conclusion, multiple heavy metals/metalloids and the soil physicochemical properties jointly shaped the diversity of the microbial communities, structure, and function in soils that were long-term contaminated with multiple heavy metals and/or metalloids. This research broadens our understanding of microbial response to long-term multiple heavy metal/metalloid contaminations. In the future, exploring the resistance mechanism of these metal-/metalloid-tolerant prokaryotes to heavy metal/metalloid would be helpful for bioremediation of heavy metal/metalloid-contaminated areas.

## Data Availability Statement

The datasets presented in this study can be found in online repositories. The names of the repository/repositories and accession number(s) can be found below: NCBI Sequence Read Archive (PRJNA728971).

## Author Contributions

XL provided the idea of this work. XH collected samples, detected and analyzed the data, prepared the figures, and wrote the manuscript. JW analyzed microbial high-throughput and prepared the figures. YL, XC, and MZ revised the manuscript. JZ extracted and detected the microbial DNA. DM, XY, and XZ analyzed the soil physicochemical and heavy metal/metalloid contents. All authors contributed to the article and approved the submitted version.

## Conflict of Interest

All authors were employed by National Engineering Laboratory of Biohydrometallurgy, GRINM Group Co., Ltd., and GRINM Resources and Environment Tech. Co., Ltd. XH and YL were employed by GRIMAT Engineering Institute Co., Ltd.

## Publisher’s Note

All claims expressed in this article are solely those of the authors and do not necessarily represent those of their affiliated organizations, or those of the publisher, the editors and the reviewers. Any product that may be evaluated in this article, or claim that may be made by its manufacturer, is not guaranteed or endorsed by the publisher.
