# Supplementary material for: Effects of Heavy Metals/Metalloids and Soil Properties on Microbial Communities in Farmland in the Vicinity of a Metals Smelter
Source: Front Microbiol. 2021 Aug 19;12:707786. doi: 10.3389/fmicb.2021.707786 (PMC8417379; doi:10.3389/fmicb.2021.707786)
Supplement: Supplementary file 1 [file Data_Sheet_1.docx]

Supplementary Material

**Table S1.**Description of each sampling site.

| Sampling site | Location (m) |
| --- | --- |
| A1 | 852 m northwest of the smelter |
| A2 | 801 m northwest of the smelter |
| A3 | 827 m northwest of the smelter |
| B1 | 1400 m northwest of the smelter |
| B2 | 1400 m northwest of the smelter |
| B3 | 1390 m northwest of the smelter |
| C1 | 2410 m northwest of the smelter |
| C2 | 2426 m northwest of the smelter |
| C3 | 2434 m northwest of the smelter |
| D1 | 2448 m southwest of the smelter |
| D2 | 2391 m southwest of the smelter |
| D3 | 2386 m southwest of the smelter |

**Table S2.**Soilphysiochemical properties and heavy metals/metalloids concentrations.

| Parameter | Site A | Site B | Site C | Site D |
| --- | --- | --- | --- | --- |
| pH | 7.30±0.15a | 5.20±0.53b | 5.07±0.13b | 4.66±0.66b |
| OM (g·kg^-1^) | 33.59±7.42a | 38.64±6.54a | 31.64±6.96a | 16.28±4.13b |
| AN (mg·kg^-1^) | 271.13±37.6a | 434.50±13.02a | 373.10±25.73a | 288.40±34.32a |
| AP (mg·kg^-1^) | 95.32±25.40a | 69.92±34.94ab | 73.9±33.41ab | 113.39±43.9a |
| AK (mg·kg^-1^) | 189.51±51.06a | 46.13±2.76c | 99.65±14.24b | 99.51±13.42b |
| T-As(mg·kg^-1^) | 59.59±15.18a | 32.49±7.09b | 24.47±0.43b | 19.50±5.28b |
| T-Cd(mg·kg^-1^) | 33.94±1.31a | 9.64±0.98b | 8.51±3.33b | 3.57±0.99b |
| T-Pb(mg·kg^-1^) | 693.38±244.67a | 385.25±37.22b | 295.79±20.81bc | 117.73±9.26cd |
| T-Zn(mg·kg^-1^) | 2702.10±853.66a | 803.38±17.28b | 559.94±64.17b | 307.53±28.45b |
| A-As(mg·kg^-1^) | 12.97±2.38a | 6.11±1.03b | 3.52±0.92c | 1.78±0.35c |
| A-Cd(mg·kg^-1^) | 10.35±3.42a | 3.05±0.24b | 3.75±1.05b | 1.05±0.37b |
| A-Pb(mg·kg^-1^) | 129.18±23.20a | 133.19±23.07a | 145.18±8.26a | 38.56±0.55b |
| A-Zn(mg·kg^-1^) | 402.95±136.62a | 111.88±7.95b | 105.31±25.49b | 43.93±13.08b |

Abbreviations: OM, Organic Matter; AN, available nitrogen; AP, available phosphorus; AK, available potassium; T-As, total concentration of arsenic; T-Cd, total concentration of cadmium; T-Pb, total concentration of lead; T-Zn, total concentration of zinc; A-As, available content of arsenic; A-Cd, available content of cadmium; A-Pb, available content of lead; A-Zn, available content of zinc; Different letters indicate significant differences between sites for that parameter.

**Table S3.** The ecology-soil selected limit values of As, Cd, Pb and Zn in agricultural soil and the values were referenced to soil environmental quality standard of China (GB 15618-2018).

|  | | pH≤5.5 | 5.5＜pH≤6.5 | 6.5＜pH≤7.5 | pH＞7.5 |
| --- | --- | --- | --- | --- | --- |
| As(mg/kg) | Paddy field | 30 | 30 | 25 | 20 |
|  | Dry land | 40 | 40 | 30 | 25 |
| Cd(mg/kg) | Paddy field | 0.3 | 0.4 | 0.6 | 0.8 |
|  | Dry land | 0.3 | 0.3 | 0.3 | 0.3 |
| Pb(mg/kg) | Paddy field | 80 | 100 | 140 | 240 |
|  | Dry land | 70 | 90 | 120 | 170 |
| Zn(mg/kg) |  | 200 | 200 | 250 | 300 |

**Table S4.**Class and pollution status of samples by comprehensive pollution indexes (*P_N_*).

| *P_N_* | class | Pollution status | Pollution level |
| --- | --- | --- | --- |
| *P_N_* ≤ 0.7 | 1 | Security | Clean |
| 0.7 <*P_N_*≤ 1 | 2 | Warning level | Still clean |
| 1<*P_N_*≤ 2 | 3 | Light | The pollutant exceeds the initial pollution value, and the crop starts to pollute |
| 2<*P_N_* ≤ 3 | 4 | Moderate | Soil and crop pollution is obvious |
| *P_N_*>3 | 5 | heavy | Serious soil and crop pollution |

**Table S5.**Class and pollution status of samples by geo-accumulation indexes (*I_geo_*).

| *I_geo_* | Class | Pollution status | Risk |
| --- | --- | --- | --- |
| < 0 | 0 | Unpolluted | No risk |
| 0-1 | 1 | Unpolluted to moderate | Low risk |
| 1-2 | 2 | Moderate | Moderate risk |
| 2-3 | 3 | Moderate to heavy | Moderate risk to high risk |
| 3-4 | 4 | Heavy | High risk |
| 4-5 | 5 | Heavy to extreme | High risk to very high risk |
| >5 | *6* | Extreme | Very high risk |

**Table S6.** Soilmicrobial community functions(%).

| functions | A | B | C | D |
| --- | --- | --- | --- | --- |
| chemoheterotrophy | 39.06±0.14 | 42.74±5.84 | 37.21±3.6 | 41.97±0.1 |
| aerobic chemoheterotrophy | 37.54±0.32 | 39.12±4.87 | 35.35±3.76 | 40.04±2.68 |
| aromatic compound degradation | 3.32±0.83 | 1.76±0.49 | 1.81±0.82 | 2.65±0.82 |
| nitrogen fixation | 1.16±0.09 | 0.75±0.06 | 2.34±0.93 | 2.04±0.99 |
| animal parasites or symbionts | 2.7±0.8 | 1.68±0.43 | 1.16±0.62 | 1.87±0.17 |
| human pathogens all | 2.68±0.81 | 1.67±0.42 | 1.16±0.61 | 1.85±0.17 |
| fermentation | 0.93±0.61 | 1.7±0.36 | 1.2±0.6 | 1.58±1.48 |
| ureolysis | 3.12±1.01 | 0.36±0.09 | 2.2±0.45 | 1.46±0.64 |
| intracellular parasites | 0.38±0.54 | 3.24±1.48 | 1.03±0.57 | 1.57±2.56 |
| nitrification | 1±0.3 | 1.28±0.59 | 0.67±0.23 | 0.75±0.31 |
| nitrate reduction | 1.11±0.27 | 0.63±0.12 | 0.9±0.14 | 0.79±0.57 |
| aerobic nitrite oxidation | 1.45±1.11 | 1.28±0.33 | 0.62±0.22 | 0.26±0.06 |
| predatory or exoparasitic | 0.7±0.35 | 1.05±0.53 | 0.54±0.29 | 0.42±0.23 |
| chitinolysis | 0.24±0.23 | 0.96±0.19 | 1.03±0.57 | 0.15±0.06 |
| phototrophy | 0.24±0.23 | 0.96±0.19 | 1.03±0.57 | 0.15±0.06 |
| photoheterotrophy | 0.32±0.2 | 0.89±0.15 | 0.94±0.37 | 0.24±0.07 |
| manganese oxidation | 0.3±0.19 | 0.87±0.15 | 0.92±0.38 | 0.23±0.06 |
| aerobic ammonia oxidation | 0.21±0.21 | 0.77±0.15 | 0.81±0.4 | 0.12±0.1 |
| nitrate respiration | 0.21±0.21 | 0.77±0.15 | 0.81±0.4 | 0.12±0.1 |
| nitrogen respiration | 0.21±0.21 | 0.77±0.15 | 0.81±0.4 | 0.12±0.1 |
| anoxygenic photoautotrophy S oxidizing | 0.2±0.22 | 0.77±0.14 | 0.8±0.4 | 0.11±0.09 |
| anoxygenic photoautotrophy | 0.19±0.2 | 0.74±0.15 | 0.8±0.4 | 0.11±0.09 |
| photoautotrophy | 0.19±0.2 | 0.74±0.15 | 0.8±0.4 | 0.11±0.09 |
| nitrite respiration | 0.19±0.2 | 0.74±0.15 | 0.8±0.4 | 0.11±0.09 |
| nitrate denitrification | 0.19±0.2 | 0.74±0.15 | 0.8±0.4 | 0.11±0.09 |
| nitrite denitrification | 0.63±0.22 | 0.42±0.14 | 0.21±0.07 | 0.31±0.04 |
| nitrous oxide denitrification | 0.3±0.13 | 0.24±0.14 | 0.13±0.1 | 0.32±0.49 |
| denitrification | 0.03±0.03 | 0.01±0.01 | 0.86±0.62 | 0.01±0.02 |
| methylotrophy | 0.03±0.01 | 0.12±0.06 | 0.43±0.43 | 0.02±0.02 |
| cellulolysis | 0.03±0.01 | 0.12±0.06 | 0.36±0.37 | 0.02±0.02 |
| hydrocarbon degradation | 0.06±0.05 | 0.15±0.09 | 0.18±0.12 | 0.04±0.03 |
| methanol oxidation | 0.04±0.06 | 0±0 | 0.33±0.02 | 0.01±0 |
| dark oxidation of sulfur compounds | 0.14±0.06 | 0.07±0.01 | 0.12±0.03 | 0.08±0.03 |
| aromatic hydrocarbon degradation | 0.38±0.41 | 0.01±0.01 | 0.08±0.06 | 0.03±0.04 |
| aliphatic nonmethane hydrocarbon degradation | 0.11±0.06 | 0.07±0.02 | 0.06±0.06 | 0.08±0.04 |
| methanotrophy | 0.08±0.04 | 0.05±0.01 | 0.04±0.02 | 0.06±0.03 |
| methanogenesis | 0.08±0.07 | 0.03±0 | 0.07±0.05 | 0.03±0.03 |
| respiration of sulfurcompounds | 0.01±0 | 0±0 | 0.15±0.18 | 0±0 |
| iron respiration | 0±0 | 0.07±0.11 | 0.05±0.06 | 0±0.01 |
| sulfate respiration | 0.03±0.01 | 0.01±0 | 0.06±0.03 | 0.02±0.03 |
| human gut | 0.03±0.05 | 0±0 | 0.07±0.06 | 0±0 |
| mammal gut | 0±0 | 0±0 | 0.08±0.07 | 0±0 |
| methanogenesis by reduction of methyl compounds with H_2_ | 0±0 | 0±0 | 0.06±0.07 | 0±0 |
| hydrogenotrophic methanogenesis | 0.05±0.06 | 0.01±0 | 0.01±0.02 | 0±0 |
| chlorate reducers | 0.05±0.06 | 0.01±0 | 0.01±0.02 | 0±0 |
| thiosulfate respiration | 0±0 | 0±0 | 0.05±0.04 | 0±0 |
| dark hydrogen oxidation | 0.02±0.02 | 0.01±0.01 | 0.01±0.01 | 0.02±0.01 |
| methanogenesis by disproportionation of methyl groups | 0.02±0.02 | 0.01±0.01 | 0.01±0.01 | 0.02±0.01 |
| methanogenesis by CO_2_ reduction with H_2_ | 0.03±0.05 | 0±0 | 0.02±0.02 | 0±0 |
| sulfur respiration | 0±0 | 0±0 | 0.01±0.02 | 0±0 |
| dark sulfide oxidation | 0±0.01 | 0±0 | 0±0 | 0±0 |
| dark sulfur oxidation | 0±0 | 0±0.01 | 0±0 | 0±0 |

**Table S7.**Spearman correlation analysis between soil environmentalvariables and microbial community functions.

|  | pH | OM | AN | AK | AP | T-As | T-Cd | T-Pb | T-Zn | A-As | A-Cd | A-Pb | A-Zn |
| --- | --- | --- | --- | --- | --- | --- | --- | --- | --- | --- | --- | --- | --- |
| chemoheterotrophy | -.636^*^ | -0.319 | 0.165 | -0.427 | 0.126 | -.587^*^ | -.846^**^ | -.699^*^ | -.783^**^ | -.734^**^ | -.888^**^ | -0.469 | -.811^**^ |
| aerobic chemoheterotrophy | -.622^*^ | -0.333 | 0.130 | -0.413 | 0.070 | -.601^*^ | -.818^**^ | -.713^**^ | -.755^**^ | -.748^**^ | -.860^**^ | -0.483 | -.797^**^ |
| aromatic compound degradation | -0.413 | -.851^**^ | -.708^*^ | 0.112 | 0.105 | -0.112 | -0.294 | -0.406 | -0.448 | -0.378 | -0.441 | -.685^*^ | -0.524 |
| ureolysis | -.706^*^ | -.588^*^ | -0.175 | -0.028 | -0.042 | -0.497 | -.594^*^ | -.699^*^ | -.797^**^ | -.741^**^ | -0.483 | -0.084 | -.615^*^ |
| nitrate reduction | -0.182 | 0.144 | 0.490 | -.678^*^ | -0.399 | 0.000 | -0.315 | -0.126 | -0.287 | -0.238 | -0.301 | 0.189 | -0.266 |
| nitrogen fixation | -0.175 | -0.340 | -0.434 | .580^*^ | 0.084 | -0.203 | 0.105 | -0.182 | -0.140 | -0.175 | 0.210 | 0.147 | 0.119 |
| chitinolysis | -0.224 | 0.263 | .771^**^ | -0.503 | 0.049 | -0.448 | -0.448 | -0.266 | -0.308 | -0.196 | -0.413 | 0.175 | -0.308 |
| nitrification | 0.077 | 0.098 | 0.284 | -.608^*^ | -0.503 | 0.154 | -0.154 | 0.007 | -0.021 | -0.035 | -0.133 | 0.133 | -0.210 |
| fermentation | 0.517 | .806^**^ | 0.452 | -0.231 | 0.035 | 0.510 | 0.357 | .615^*^ | 0.524 | 0.483 | 0.273 | 0.175 | 0.476 |
| aerobic nitrite oxidation | 0.259 | 0.308 | .585^*^ | -0.462 | -0.441 | 0.000 | 0.133 | 0.161 | 0.259 | 0.273 | 0.140 | 0.392 | 0.140 |
| nitrate respiration | -0.091 | 0.322 | .806^**^ | -0.531 | -0.462 | -0.210 | -0.077 | -0.084 | -0.140 | -0.084 | -0.063 | 0.490 | -0.042 |
| nitrogen respiration | -0.091 | 0.322 | .806^**^ | -0.531 | -0.462 | -0.210 | -0.077 | -0.084 | -0.140 | -0.084 | -0.063 | 0.490 | -0.042 |
| phototrophy | -0.091 | 0.343 | .813^**^ | -0.566 | -0.448 | -0.161 | -0.112 | -0.063 | -0.161 | -0.084 | -0.084 | 0.510 | -0.063 |
| photoheterotrophy | -0.091 | 0.343 | .813^**^ | -0.566 | -0.448 | -0.161 | -0.112 | -0.063 | -0.161 | -0.084 | -0.084 | 0.510 | -0.063 |
| anoxygenic photoautotrophy | -0.049 | 0.329 | .827^**^ | -0.469 | -0.427 | -0.224 | -0.007 | -0.042 | -0.091 | -0.007 | -0.014 | 0.517 | 0.028 |
| photoautotrophy | -0.049 | 0.329 | .827^**^ | -0.469 | -0.427 | -0.224 | -0.007 | -0.042 | -0.091 | -0.007 | -0.014 | 0.517 | 0.028 |
| anoxygenic photoautotrophy S oxidizing | -0.049 | 0.329 | .827^**^ | -0.469 | -0.427 | -0.224 | -0.007 | -0.042 | -0.091 | -0.007 | -0.014 | 0.517 | 0.028 |
| nitrite respiration | -0.049 | 0.329 | .827^**^ | -0.469 | -0.427 | -0.224 | -0.007 | -0.042 | -0.091 | -0.007 | -0.014 | 0.517 | 0.028 |
| nitrate denitrification | -0.049 | 0.329 | .827^**^ | -0.469 | -0.427 | -0.224 | -0.007 | -0.042 | -0.091 | -0.007 | -0.014 | 0.517 | 0.028 |
| nitrite denitrification | -0.049 | 0.329 | .827^**^ | -0.469 | -0.427 | -0.224 | -0.007 | -0.042 | -0.091 | -0.007 | -0.014 | 0.517 | 0.028 |
| nitrous oxide denitrification | -0.049 | 0.329 | .827^**^ | -0.469 | -0.427 | -0.224 | -0.007 | -0.042 | -0.091 | -0.007 | -0.014 | 0.517 | 0.028 |
| denitrification | -0.049 | 0.329 | .827^**^ | -0.469 | -0.427 | -0.224 | -0.007 | -0.042 | -0.091 | -0.007 | -0.014 | 0.517 | 0.028 |
| iron respiration | -0.011 | 0.067 | 0.210 | 0.226 | -0.190 | -0.145 | 0.159 | 0.018 | 0.004 | 0.085 | 0.339 | .646^*^ | 0.229 |
| respiration of sulfurcompounds | -0.028 | 0.491 | .800^**^ | -0.357 | -0.249 | -0.221 | -0.053 | -0.004 | -0.028 | -0.004 | 0.091 | .669^*^ | 0.088 |
| sulfate respiration | -0.046 | 0.486 | .821^**^ | -0.441 | -0.322 | -0.217 | -0.056 | -0.004 | -0.014 | -0.007 | 0.098 | .701^*^ | 0.088 |
| manganese oxidation | 0.308 | -0.091 | -.596^*^ | .666^*^ | 0.011 | 0.301 | 0.483 | 0.287 | 0.368 | 0.277 | 0.522 | 0.053 | 0.399 |

* indicates 0.01 < p ≤ 0.05, ** indicates 0.001 < p ≤ 0.01, *** indicates p ≤ 0.001.

**Table S8.**Spearman correlation analysis between soil environmentalParameters.

|  | A-As/T-As | A-Cd/T-Cd | A-Pb/T-Pb | A-Zn/T-Zn |
| --- | --- | --- | --- | --- |
| pH | 0.825^**^ | -0.014 | -0.441 | -0.406 |
| OM | 0.490 | 0.354 | -0.011 | -0.231 |
| AN | 0.168 | 0.361 | 0.476 | 0.060 |
| AK | 0.077 | 0.007 | -0.350 | 0.273 |
| AP | -0.350 | -0.147 | -0.196 | 0.042 |

* indicates 0.01 < p ≤ 0.05, ** indicates 0.001 < p ≤ 0.01, *** indicates p ≤ 0.001.

**Table S9.**Spearman correlation analysis between soil environmentalparameters, comprehensive pollution index and geo-accumulation index (I_geo_) and bacterial alpha diversity indexes.

| Parameters | richness index | | Shannon index | |
| --- | --- | --- | --- | --- |
|  | *R* | *P* | *R* | *P* |
| Comprehensive pollution index | 0.098 | 0.762 | 0.168 | 0.602 |
| I_geo_(T-As) | 0.056 | 0.863 | 0.301 | 0.342 |
| I_geo_(T-Cd) | 0.056 | 0.863 | 0.154 | 0.633 |
| I_geo_(T-Pb) | 0.510 | 0.090 | 0.559 | 0.059 |
| I_geo_(T-Zn) | 0.175 | 0.587 | 0.294 | 0.354 |
| pH | 0.147 | 0.649 | 0.280 | 0.379 |
| OM (g·kg^-1^) | .609^*^ | 0.035 | 0.508 | 0.092 |
| AN (mg·kg^-1^) | .774^**^ | 0.003 | .627^*^ | 0.029 |
| AP (mg·kg^-1^) | -.650^*^ | 0.022 | -.741^**^ | 0.006 |
| AK (mg·kg^-1^) | -0.559 | 0.059 | -.692^*^ | 0.013 |
| T-As(mg·kg^-1^) | 0.056 | 0.863 | 0.301 | 0.342 |
| T-Cd(mg·kg^-1^) | 0.056 | 0.863 | 0.154 | 0.633 |
| T-Pb(mg·kg^-1^) | 0.196 | 0.542 | 0.3 50 | 0.265 |
| T-Zn(mg·kg^-1^) | 0.175 | 0.587 | 0.294 | 0.354 |
| A-As(mg·kg^-1^) | 0.098 | 0.762 | 0.259 | 0.417 |
| A-Cd(mg·kg^-1^) | 0.140 | 0.665 | 0.196 | 0.542 |
| A-Pb(mg·kg^-1^) | .629^*^ | 0.028 | .594^*^ | 0.042 |
| A-Zn(mg·kg^-1^) | 0.189 | 0.557 | 0.231 | 0.471 |

* indicates 0.01 < p ≤ 0.05, ** indicates 0.001 < p ≤ 0.01, *** indicates p ≤ 0.001.


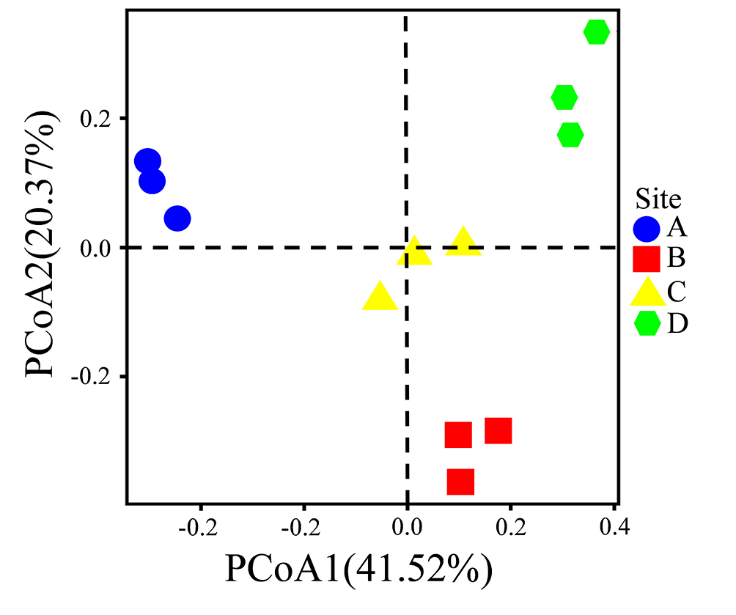


**Figure S1.**A principal coordinates analysis (PCoA) plot depicting the microbial community composition (OTUs) in soil samples from different sampling sites, based on Euclidean distances.


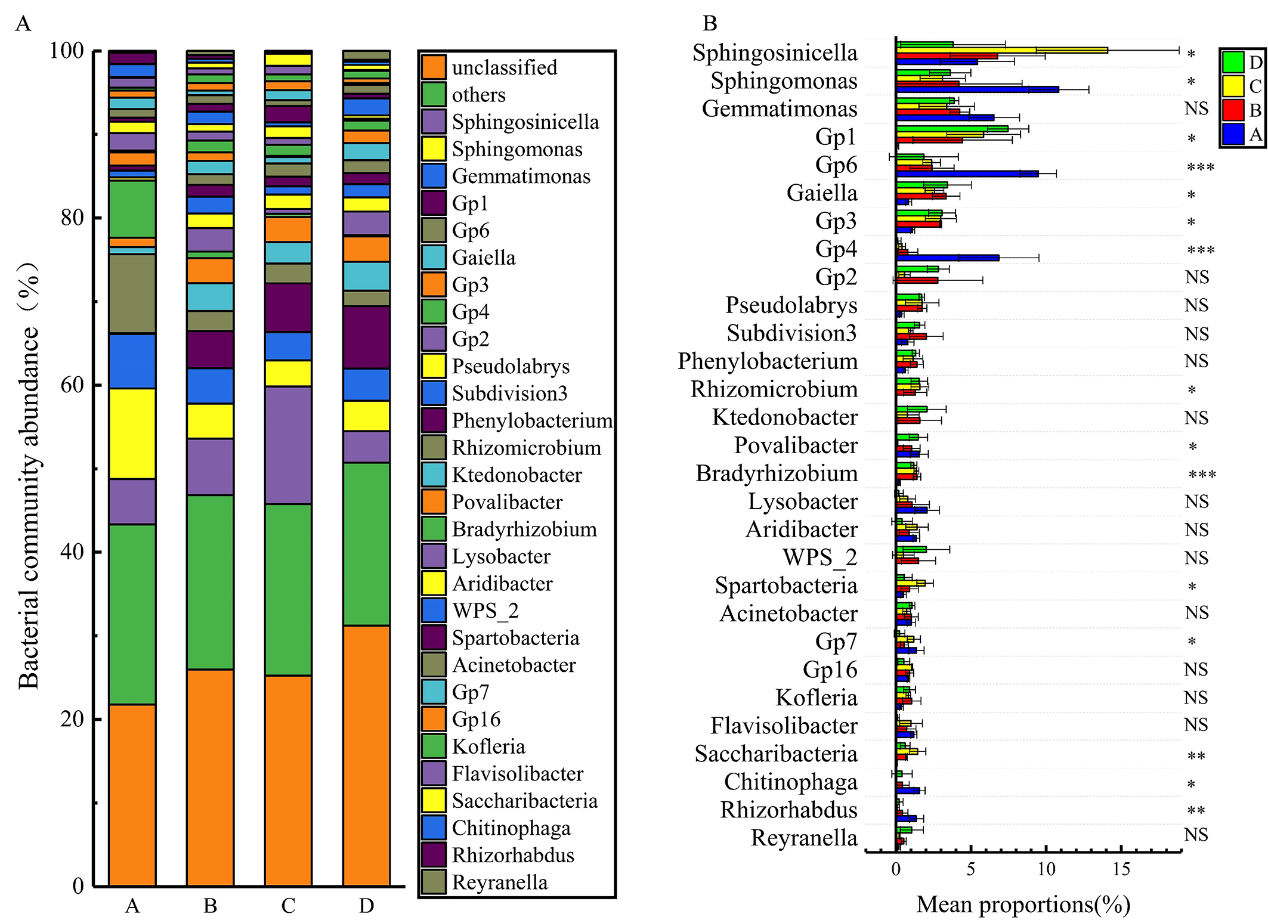


**Figure S2.**Abundance of bacterial community in four sites Soil bacterial (a) community composition and (b) relative abundance differences at genus levels. Only the top 30 most abundant genus are showed. A one-way ANOVA is used to evaluate relative abundance differences between the four sites (* indicates 0.01 < p ≤ 0.05, ** indicates 0.001 < p ≤ 0.01, *** indicates p ≤ 0.001, and NS, indicates not significant).


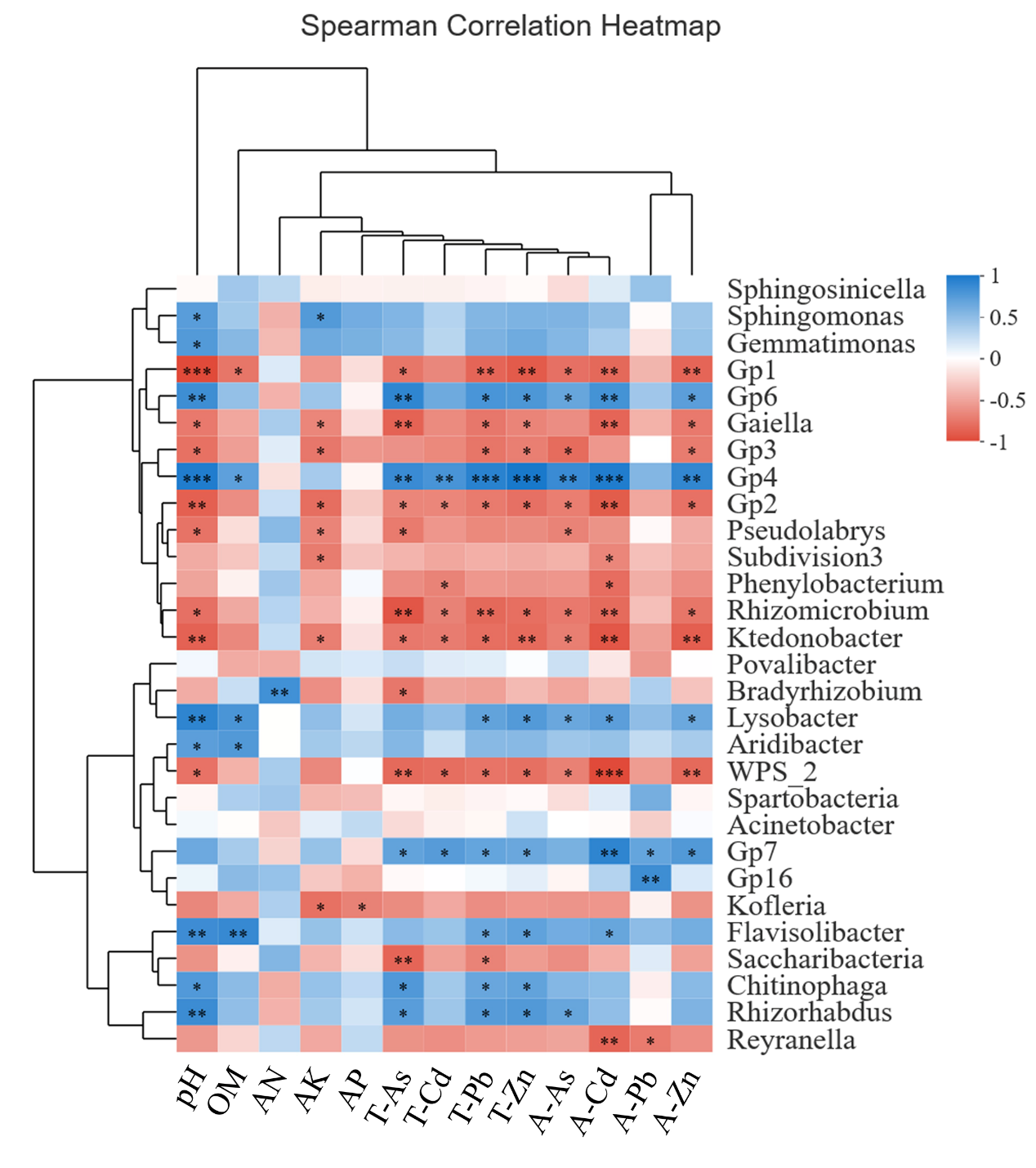


**Figure S3.** Heatmap of Spearman's rank correlation coefficients between the four sites the relative abundances of bacterial genus, including the relative abundances (%, n=3) of the top 30 microbial genera. Note: Horizontal row represents soil physiochemical properties and metals/metalloids information, vertical row represents microbial community abundance information, red represents negative correlation, blue represents positive correlation, darker color indicates higher correlation, p value is correlation test result, * in the figure indicates p < 0.05, and ** indicates p < 0.01. OM, Organic Matter; AN, available nitrogen; AP, available phosphorus; AK, available potassium; T-As, total concentration of arsenic; T-Cd, total concentration of cadmium; T-Pb, total concentration of lead; T-Zn, total concentration of zinc; A-As, available content of arsenic; A-Cd, available content of cadmium; A-Pb, available content of lead; A-Zn, available content of zinc.
